# Global assessment of migraine severity measure: preliminary evidence of construct validity

**DOI:** 10.1186/s12883-019-1284-8

**Published:** 2019-04-04

**Authors:** Tolulope T. Sajobi, Farnaz Amoozegar, Meng Wang, Natalie Wiebe, Kirsten M. Fiest, Scott B. Patten, Nathalie Jette

**Affiliations:** 1 Department of Community Health Sciences and O’Brien Institute for Public Health, University of Calgary, Cumming School of Medicine 3280 Hospital Drive NW Calgary, Calgary, Alberta T2N 4Z6 Canada; 2 Department of Clinical Neurosciences, University of Calgary, Calgary, Canada; 3 Department of Critical Medicine, University of Calgary, Calgary, Canada; 4 Department of Psychiatry, University of Calgary, Calgary, Canada; 5 Department of Neurology, Icahn School of Medicine at Mount Sinai, New York, USA

**Keywords:** Migraine severity, Self-report, Global assessment of migraine severity, Depression, Disability, Construct validity

## Abstract

**Background:**

In persons with migraine, severity of migraine is an important determinant of several health outcomes (e.g., patient quality of life and health care resource utilization). This study investigated how migraine patients rate the severity of their disease and how these ratings correlate with their socio-demographic, clinical, and psycho-social characteristics.

**Methods:**

This is a cohort of 263 adult migraine patients consecutively enrolled in the Neurological Disease and Depression Study (NEEDs). We obtained a broad range of clinical and patient-reported measures (e.g., patients’ ratings of migraine severity using the Global Assessment of Migraine Severity (GAMS), and migraine-related disability, as measured by the Migraine Disability Scale (MIDAS)). Depression was measured using the 9-item Patient Health Questionnaire (PHQ-9) and the 14-item Hospital Anxiety and Depression Scale (HADS). Median regression analysis was used to examine the predictors of patient ratings of migraine severity.

**Results:**

The mean age for the patients was 42.5 years (SD = 13.2). While 209 (79.4%) patients were females, 177 (67.4%) participants reported “moderately severe” to “extremely severe” migraine on the GAMS, and 100 (31.6%) patients had chronic migraine. Patients’ report of severity on the GAMS was strongly correlated with patients’ ratings of MIDAS global severity question, overall MIDAS score, migraine type, PHQ-9 score, and frequency of migraine attacks. Mediation analyses revealed that MIDAS mediated the effect of depression on patient ratings of migraine severity, accounting for about 32% of the total effect of depression. Overall, migraine subtype, frequency of migraine, employment status, depression, and migraine-related disability were statistically significant predictors of patient-ratings of migraine severity.

**Conclusions:**

This study highlights the impact of clinical and psychosocial determinants of patient-ratings of migraine severity. GAMS is a brief and valid tool that can be used to assess migraine severity in busy clinical settings.

## Background

Migraine is a neurological condition characterized by recurrent headaches and other neurological symptoms. Migraine represents one-third of all neurological disease burden [1] and is one of the top 15 conditions with the most substantially increased disease burden ranking in the past decade; it is among the top 25 causes of years lived with disability (YLDs)^2^. Migraine leads the list of neurological disorders, representing more than 50% of neurological YLDs or 22.9% of global YLDs [2], imposing considerable burden on the migraineurs and on society. Approximately 90% of persons with migraine have moderate or severe pain, three-quarters have reduced ability to function during headache attacks, and one-third require bed rest during their attacks [3–6].

Several clinical and epidemiological studies have recognized migraine severity as an important outcome when assessing treatment efficacy in the management of migraine [7–10]. Migraine headaches are associated with substantial functional impairment, reduced health-related quality of life, and psychiatric comorbidities [10–12]. Several standardized and validated scales have been developed to assess patient-reported or physician-reported migraine severity, a concept closely linked to disability [13–16]. These include the Migraine Disability Assessment Scale (MIDAS) [11], the Headache Impact test [14], the Henry Ford Disability Inventory [15], and the Migraine Severity Scale [16]. However, the majority of these measures are focused on migraine-related disability and are sensitive to recall bias. In addition, brief measures of patients’ perspectives regarding their disease severity, as distinct from clinician’s views, are especially needed in busy clinical settings. One possibility is to use a single item, self-defined global QOL measure to address a patient’s subjective, self-rated measure of severity. This type of a global measure would incorporate all aspects of the disease evaluated in one single item.

The limitations of longer severity measures are addressed in a brief single item patient-reported measure, the Global Assessment of Migraine Severity (GAMS), developed to assess patients’ perception of their disease severity. The objectives of this current study were to (1) explore how patients with migraine perceive the severity of their disease and its inter-relationship with patients’ demographic, clinical, and psychosocial characteristics, and (2) assess how GAMS compares to other measures of migraine severity and disability. We hypothesize that patients’ ratings of migraine severity would be strongly correlated with other validated measures of migraine severity and patients’ psychosocial characteristics.

## Methods

### Study design

Data for this study were prospectively obtained through the NEurological diseasE and Depression Study (NEEDs), a cross-sectional study that investigates the prevalence of depression in several cohorts of patients with neurological conditions [17, 18]. The migraine cohort consisted of 263 consecutive migraine patients seen in Calgary, Canada (catchment area > 1 million people) between 2012 and January 2013. Patients 18 years of age and older with a neurologist reported diagnosis of migraine according to the third edition of the International Classification of Headache Disorders criteria were included. Patients also had to speak and read English fluently, have no hearing impairment, and have no specialist-diagnosed dementia, moderate or severe developmental delay, or aphasia. Data collected included socio-demographic characteristics such as employment status, age, sex, educational status, and marital status. Data were collected on relevant clinical characteristics, such as migraine frequency (i.e., episodic versus chronic migraine), migraine subtype (with aura versus without aura), number of years since migraine onset, self-reported side effects from medication (yes or no). In addition to these data elements, patient-reported data were collected as described below:

#### Migraine disability assessment (MIDAS)

The MIDAS was developed to assess headache-related disability with the aim of improving migraine care [13]. It is a self-administered questionnaire designed to quantify headache-related disability over a 3-month period [19]. This questionnaire consists of five questions that focus on time or productivity lost, as well as the limited ability to participate in work or school, household activities and family, and social or leisurely activities. The total MIDAS score can be used to define four grades of migraine-related disability with grade I for “little or no disability” (0–5); grade II for “mild disability” (6–10); grade III for “moderate disability” (11–20); and grade IV for “severe disability” (≥ 21). The MIDAS also includes a migraine severity global question, with subjects’ responses ranging between 0 (no pain at all) to 10 (very severe pain). Two additional questions included in the MIDAS provide the physician with supplementary clinical information about headache frequency and severity/intensity (scale from 0 to 10) of headaches over the previous three months. The MIDAS is a reliable and valid instrument with moderately high test-retest reliability in persons with migraine and correlates to clinical judgment regarding the need for medical care [20].

#### Patient health questionnaire

The Patient Health Questionnaire (PHQ-9) is a validated 9-item self-reported questionnaire for screening, diagnosing, monitoring, and measuring the severity of depression [21, 22]. The PHQ-9 consists of the nine DSM-IV criteria scored on a 4-point Likert scale from “0” (not at all) to “3” (nearly every day). The PHQ-9 scale yields a total score ranging from 0 to 27. Scores of 5, 10, 15, and 20 represent mild, moderate, moderately severe, and severe depression, respectively. The PHQ-9 has recently been validated in patients with migraine including in our cohort [23, 24].

#### Hospital anxiety and depression scale

The HADS is a 14-item screening tool for depression and anxiety developed for use in populations with medical conditions [25]. It includes 7 items for the anxiety (HADS-A) subscale and 7 items for the depression (HADS-D) subscale scored on a 4-point Likert scale (0–3) but only the anxiety subcomponent was used for this study. The ratings are summed up to give a score ranging from 0 (no symptoms) to 21 (maximum distress) for each subscale. The HADS has been previously validated in migraine patients, including in this cohort [24, 26, 27]. Consistent with the HADS manual’s definition of varying degrees of severity of depression and anxiety [24], we dichotomize HADS-A into a binary variable using a cutoff of 10, thereby distinguishing patients with normal/mild symptoms from those with moderate/severe symptoms of anxiety.

#### Global assessment of migraine severity (GAMS)

An assessment of severity related directly to migraine was obtained by asking the patient to respond to a single question with seven response categories: “Taking into account all aspects of your disease, how severe is your disease?” (1) Not at all severe, (2) a little severe, (3) somewhat severe (4) moderately severe, (5) quite severe, (6) very severe, (7) extremely severe. This single item severity question measures the overall severity since migraine onset. Although this item was originally developed and validated for epilepsy patients [28, 29], this measure was adapted and administered to our migraine population because of the perception that a global rating scale could be valuable, both in terms of its brevity and also in terms of its clinical relevance as a global patient-centered indicator. The format of the single item GAMS and the choice of response options were based on those of the previously validated Global Assessment of Severity in Epilepsy (GASE), a widely used single item measure of severity in epilepsy [28].

The study was approved by the University of Calgary Conjoint Health Research Ethics Board.

### Statistical analysis

Means and standard deviations were used to summarize continuous variables while frequency distributions were used to summarize discrete outcomes. Specifically, Fisher’s exact test was used to assess the association between patients’ ratings on GAMS and sex, marital status, employment status, education, medication side effects, migraine subtype, MIDAS (No/little/mild vs moderate/severe). Similar descriptive analyses were conducted separately for patients with episodic and chronic migraine. We evaluated the validity of the GAMS using correlation analysis. Specifically, the association between patients’ ratings on the GAMS and MIDAS, PHQ9, HADS-D, HADS-A, and migraine frequency was assessed using polyserial correlation. Associations between patients’ ratings on the GAMS and binary or ordinal clinical and self-reported characteristics (e.g., frequency of migraine attacks, intensity of migraine pain, migraine subtype, use of psychotropic medications, side-effects from medication, employment status) were assessed using biserial correlations. Median regression analysis was used to model the association between influence of migraine disability assessment, socio-demographic characteristics (sex, age, employment status, education and marital status), self-reported side effects from medication (yes or no), migraine subtype, migraine frequency classification, and self-reported depression and anxiety symptoms (PHQ-9).

Mediation analysis based on median regression [30] was used to assess the mediating effect of migraine-related disability on the association between severity and model predictors. When the assumptions of mediation analysis are satisfied, the extent to which the relationships between GAMS (severity) and the clinical, socio-demographic, and self-reported factors were mediated by MIDAS was expressed as a percentage. Sobel was used to assess the statistical significance of the mediation effects. Mediation analysis was conducted using the “mediation” package in R software. All analyses were conducted using R software [31].

## Results

### Patients’ characteristics

**Fig. 1:**
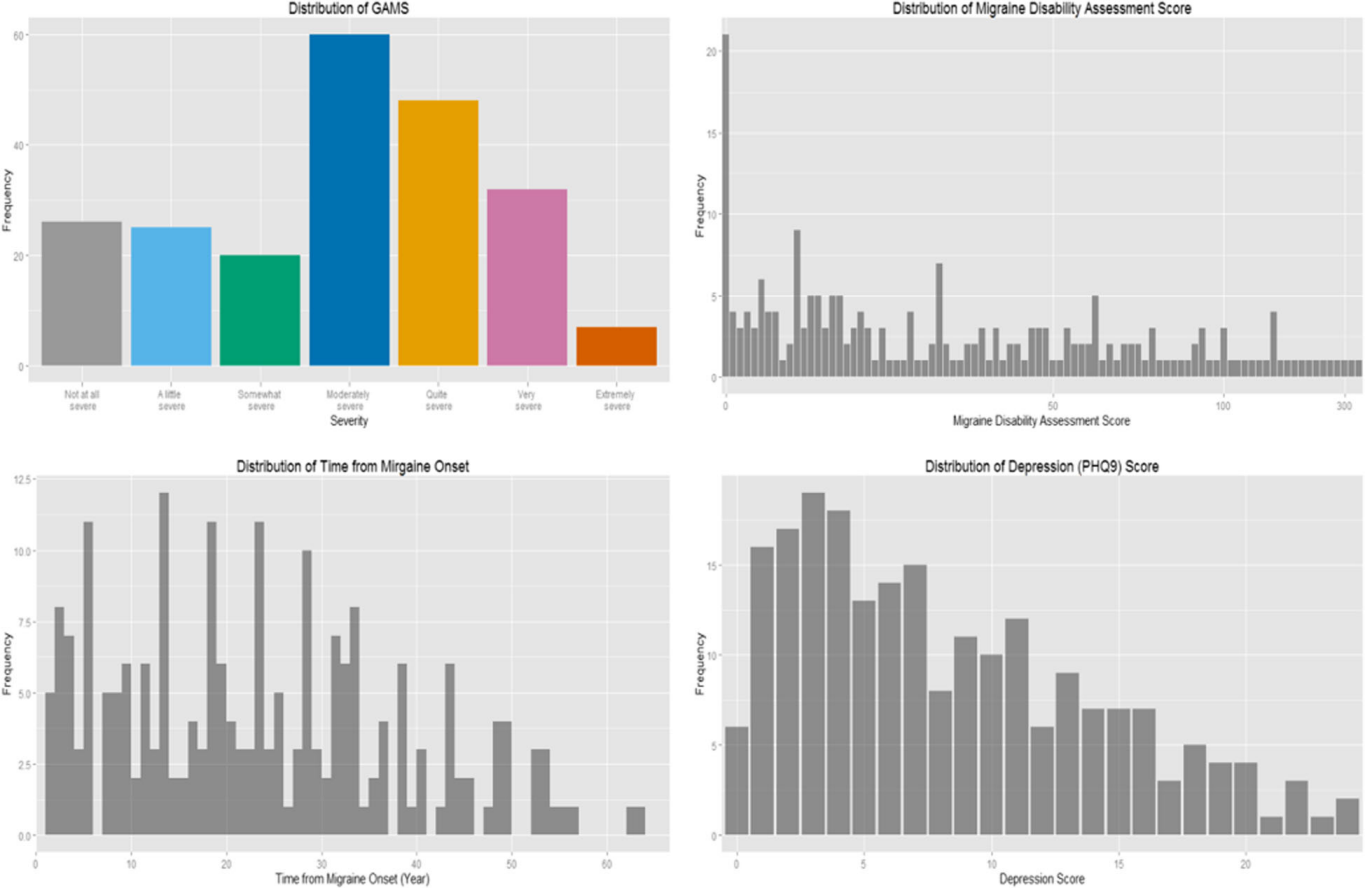
Distributions of GAMS, MIDAS and PHQ-9 Scores NB: GAMS = Global Assessment of Migraine Severity; MIDAS = Migraine Disability Scale; PHQ-9 = 9-item Patient Health Questionnaire

Table 1 describe patients’ socio-demographic, clinical, and psychosocial characteristics. Of the 263 participants, 79.4% were females, 66.5% were married or in a common-law relationship, and the mean age was 42.5 (SD = 13.2) years. Patients’ median and mean ratings of migraine severity, as measured by GAMS, were 4 and 3.93, respectively. 23.4% of the patients endorsed “not at all”, “severe” or “a little severe” migraine, 11.0% endorsed “somewhat severe” migraine, 26.6% endorsed “moderately severe” migraine, 19.8% endorsed “quite severe” migraine, 16.3% endorsed “very severe” migraine, while only 3% of the patients endorsed “extremely severe” migraine. The mean number of migraine attacks reported by the patients per month was 12.4 (SD = 10.83), while the mean MIDAS score was 46.12 (SD = 62.54), with 18% of the patients reporting little or no migraine disability, and 55.5% of the patients reported severe migraine-related disability. Moreover, 33.8% of the patients endorsed clinically elevated levels of significant depression symptoms (i.e., PHQ-9 score ≥ 10), while 30.4% of the patients endorsed clinically elevated levels of anxiety symptoms (HADS-A ≥ 10). Figure 1 shows the distribution of GAMS, MIDAS score, time from migraine onset, and PHQ-9 score. There were no significant demographic differences between patients with episodic and chronic migraine. But patients with chronic migraine rated their migraine to be generally more severe (*p* < 0.01) and more disabling (*p* < 0.01) than patients with episodic migraine. The former group is less likely to have paid employment (*p* < 0.01) but endorse more depression symptoms (*p* < 0.01) than the latter.

**Table 1:** Demographic and Clinical Characteristics of Study Participants

Characteristics	Total Sample	Episodic (*N* = 163)	Chronic (*N* = 100)	*P*-values
Age in years (mean, SD)	42.5(13.2)	43.2 (12.1)	41.3 (14.8)	0.12
Gender (*n*, %)				0.75
*Male*	51(20.6%)	33 (20.3)	18 (18.0)	
*Female*	212(79.4%)	130 (79.8)	82 (82.0)	
Marital Status (n, %)				0.18
*Married/Common Law*	179(68.1%)	116 (71.2)	63 (63.0)	
*Single/Widowed/Separated/Divorced*	84(31.9%)	47 (28.8)	37 (37.0)	
Employment Status (n, %employed)	167(65.8%)	117 (71.8)	50 (50.0)	< 0.01
Education (n, %, Bachelors)	156(59.3%)	94 (57.5)	62 (62.0)	0.52
Medication Side Effects (Yes, n, %)	125(47.5%)	72 (44.2)	53 (53.0)	0.20
Migraine subtype (n, %aura)	169 (64.3%)	106 (65.0)	63 (63.0)	0.79
Use of Psychotropic Medication (n, %)	96(36.5%)	40(40.0%)	56(34.65)	0.43
MIDAS (n, %)				< 0.01
*No/ Little*	48(18.3%)	33 (20.3)	15 (15.0)	
*Mild*	25(9.5%)	19 (11.7)	6 (6.0)	
*Moderate*	44(16.7%)	35 (21.5)	9 (9.0)	
*Severe*	146(55.5%)	76 (46.6)	70 (70.0)	
GAMS (n, %)				< 0.01
*Not at all severe*	31(11.8%)	26 (16.0)	5 (5.0)	
*A little severe*	30(11.4%)	25 (15.3)	5 (5.0)	
*Somewhat severe*	29(11.0%)	21 (12.9)	8 (8.0)	
*Moderately severe*	70(26.6%)	44 (27.0)	26 (26.0)	
*Quite severe*	52(19.8%)	30 (18.4)	22 (22.0)	
*Very severe*	43(16.3%)	16 (9.8)	27 (27.0)	
*Extremely severe*	8(3.0%)	1 (0.6)	7 (7.0)	
HADS-Anxiety (mean, SD)	7.6 (4.4)	7.2 (4.3)	8.2 (4.5)	0.07
HADS-Depression (mean, SD)	5.3 (4.1)	4.6 (4.0)	6.4 (4.2)	< 0.01
PHQ9 (mean, SD)	7.9 (6.1)	7.0 (5.9)	9.3 (6.2)	< 0.01

*SD* Standard deviation, *MIDAS* Migraine disability assessment scale, *HADS* Hospital Anxiety and Depression Scale, *GAMS* Global Assessment of Migraine Severity, P-values were based on Fisher exact tests and Wilcoxon-Mann-Whitney tests for categorical and continuous variables, respectively

### Validity of GAMS

**Fig. 2:**
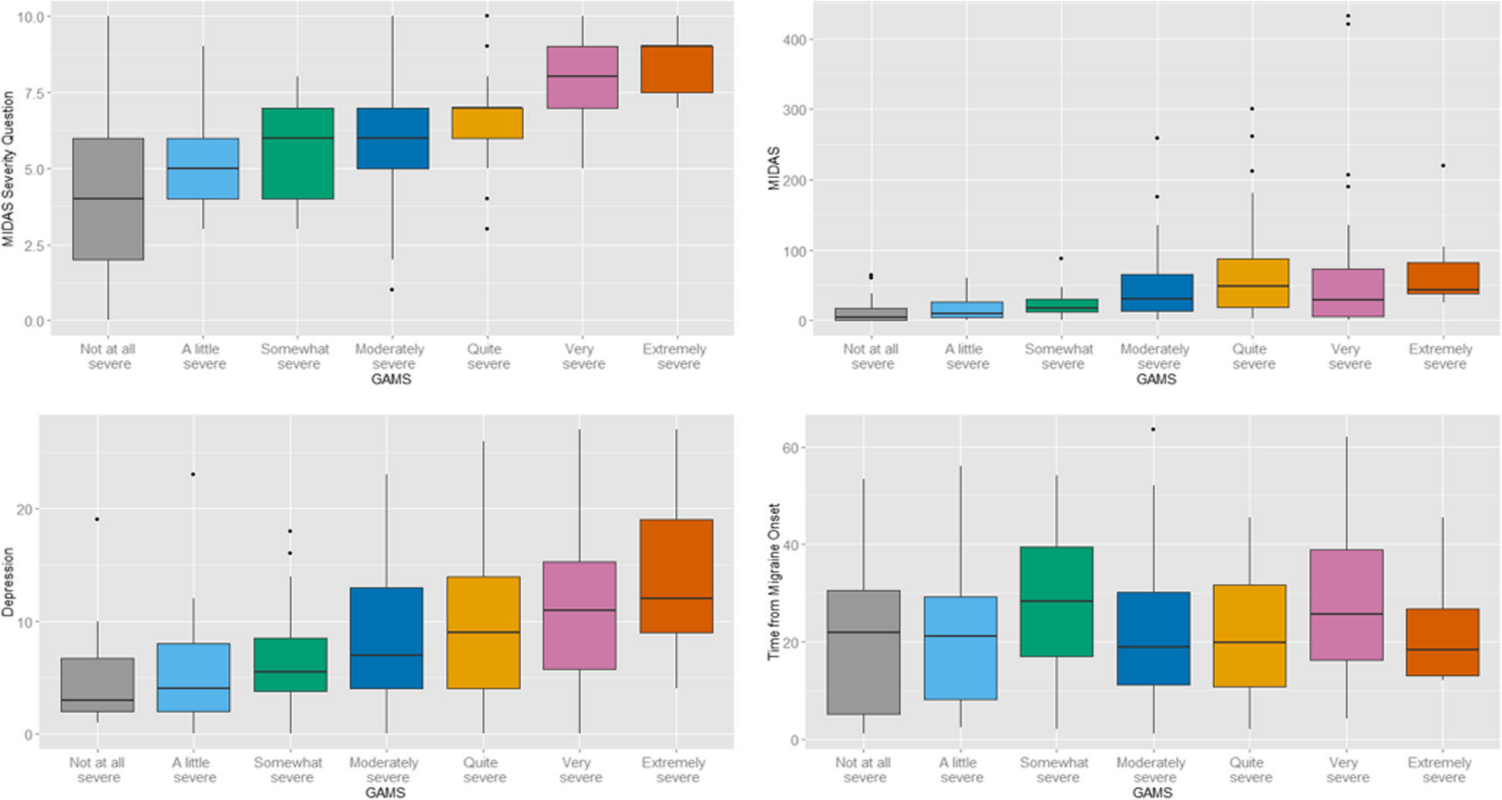
Association between GAMS and MIDAS Severity Question, MIDAS, PHQ-9 score and Time from Migraine Onset NB: GAMS = Global Assessment of Migraine Severity; MIDAS = Migraine Disability Scale; PHQ-9 = 9-item Patient Health Questionnaire

Pearson’s chi square test revealed statistically significant univariate associations between patients’ ratings of migraine severity, as measured by GAMS, and patients’ rating on migraine-related disability, migraine subtype (chronic versus episodic), and employment status (employed versus not employed). There were moderate to strong univariate correlations between patients’ ratings of migraine severity on the GAMS and depression (*r* = 0.59; *p* < 0.01), anxiety (*r* = 0.48; *p* < 0.01), frequency of migraine attacks (*r* = 0.58; p < 0.01), migraine type (*p* < 0.01), and headache intensity (*r* = 0.34; *p* < 0.05), and migraine subtype (*r* = 0.43; *p* < 0.01) (Fig. 2). However, patients’ ratings on the GAMS were not significantly associated with education, marital status, sex, use of psychotropic medications, self-reported medication side effects or aura.

### Predictors of patient-ratings of migraine severity (GAMS)

Table 2 describes the results of the median regression analysis, which models the association between patients’ ratings of migraine severity and their demographic, clinical, and psychosocial characteristics. Migraine subtype, frequency of migraine, employment status, depression, and migraine-related disability were all statistically significant predictors of patients’ ratings of migraine severity. That is, patients who report more severe migraines are likely to be unemployed individuals who report more disabling, chronic migraine with aura, and endorse clinically elevated levels of depression symptoms. Specifically, patients who endorse more symptoms of depression are likely to report a 0.471 unit increase on the GAMS than less depressed patients, while employed patients are likely to report a 0.681 unit decrease on the GAMS than unemployed patients. Patients with migraine aura are likely to report a 0.405 unit increase on the GAMS than migraine patients without aura, while patients with chronic migraine are likely to report a 0.758 unit increase on the GAMS than patients with episodic migraine.

**Table 2:** Association between GAMS and Patients’ Demographic, Clinical and Self- Reported Characteristics with MIDAS as the mediation variable

Patients’ Characteristics	Regression Coefficients	95% CI
Age	−0.004	(−0.020, 0.007)
Sex (*Female* vs. *Male*)	−0.196	(−0.888, 0.329)
Marital Status (*Not married* vs. *Married*)	0.499	(− 0.150, 1.036)
Education (*Bachelor’s Degree or above* vs. *below*)	−0.098	(− 0.376, 0.171)
Employment Status (*Paid Job* vs. *Not Paid Job*)	−0.681^*^	(− 0.949, − 0.124)
Medication Side Effects (*Yes* vs. *No*)	0.001^*^	(−0.327, 0.448)
Migraine Type (*Without Aura* vs. *With Aura*)	0.405^*^	(0.093, 0.869)
Migraine Frequency (*Chronic* vs. *Episodic*)	0.758^*^	(0.505, 1.053)
Depression (PHQ-9) (*Yes* vs. *No*)	0.471^*^	(0.208, 0.872)
Anxiety (HADS-Anxiety) (*Yes* vs. *No*)	0.316	(−0.189, 0.773)
MIDAS (Migraine Disability Assessment Scale)	0.004^*^	(< 0.001, 0.005)

^*^p < 0.05; *GAMS* Global Assessment of Migraine Severity, *PHQ-9* 9-item Patient Health Questionnaire, *HADS* Hospital Anxiety Depression Scale

Mediation analysis revealed that migraine-related disability (measured by MIDAS) partially mediated the effects of depression and employment status on patient-reported GAMS ratings. More specifically, the indirect effects of depression that were partially mediated through migraine-related disability (MIDAS), accounted for 24.8% of the total effect of depression on severity of migraine. Overall, patients with clinically elevated levels of depression symptoms were likely to self-report a 0.63 unit increase on the GAM, after adjusting for the indirect effects through migraine-related disability as measured by MIDAS, as compared to an unadjusted effect of 0.47. On the other hand, the indirect effects of depression mediated through the MIDAS accounted for 9.5% of the employment status patients’ ratings of migraine severity (measured by GAMS). After adjusting for the indirect effects through MIDAS score, unemployed patients were likely to report a 0.75 unit increase on the GAMS than employed individuals.

## Discussion

In our headache outpatient clinic, we investigated the validity of GAMS, a brief patient-reported measure of migraine severity in migraine patients, and explored the determinants of patient-ratings of migraine severity in this outpatient cohort. Our study demonstrates a moderate to strong correlation between patient-ratings of migraine severity and clinical characteristics and validated measures such as MIDAS score, MIDAS migraine severity question, PHQ9, HADS-A, migraine subtype, frequency of migraine attacks. Moreover, patients who self-report more severe migraines are more likely to be unemployed, endorse more depression symptoms, and report more disabling, chronic migraine with aura. We also identified a mediating effect of migraine disability on the determinants of migraine severity in this patient population.

Our analyses revealed that patients with elevated levels of depression symptoms were more likely to report a higher GAMS score compared with individuals with less depression symptoms. In fact, about 31.1% of patients in our cohort endorsed elevated levels of depression symptoms in the past 2 weeks (i.e., PHQ-9 ≥ 10) while 36.5% of the patients were on depression-related treatments (e.g., psychotropic medication), which are almost twice the lifetime prevalence of major depression among community dwelling persons with migraine in Canada [32], acknowledging that patients with elevated depressive symptoms comprise a broader concept of depression than that of a diagnostic definition [33].This finding is consistent with earlier studies that highlight the burden of depression in persons with migraine [24, 27, 34, 35]. For example, persons with migraine are known to have higher lifetime prevalence of psychiatric comorbidities such as major depressive disorders, panic disorders, generalized anxiety disorder, and suicidal attempts than those without migraine [34, 35]. While it can be easily argued that a severity measure should not include depression since that is a comorbid condition that would be targeted apart from the migraine symptoms, this finding highlights the interrelationship between depression and migraine, which has been noted in several studies of individuals with migraine [36–38]. This knowledge of the inter-relationship between depression and migraine can aid the design and implementation of targeted interventions for addressing depression in persons with migraine.

Our study identified employment as a statistically significant predictor of patients’ ratings in GAMS. Individuals who self-report higher migraine severity were likely to be without paid jobs. This finding is consistent with previous studies that suggest the economic burden of migraine falls predominantly on patients and their employers in the form of bedridden days and lost productivity [39–41]. Migraine frequency was also a statistically significant predictor of GAMS in the current study. Individuals with chronic migraine were more likely to report a higher severity level compared with individuals with episodic migraine, which is consistent with findings from prior studies examining the impact of chronic versus episodic migraine [7].

### Strengths

The novelty of our study finding lies in the use of a single item global rating of migraine severity to identify clinical and psychosocial determinants of patient-reported severity of migraine. In addition, the aim of GAMS is to obtain a global statement directly from patients about their perception of migraine severity. For this global statement, the item response choices are formatted directly after commonly used seven-point Likert scales in other instruments to be able to capture small but important differences. Moreover, the brevity of the GAMS makes it an ideal tool to assess severity of migraine in busy clinical settings.

### Limitations

This study has some limitations. First, although this study provides preliminary evidence about the validity of the patient-ratings on the GAMS in relation to established measures such as the MIDAS, we could not assess the reliability of GAMs because of the cross-sectional nature of this study’s design. We could only establish the construct validity of the GAM questionnaire in this cross-sectional study. A future prospective longitudinal study to assess the reliability and other psychometric properties of the GAMS in relation to other validated scales such as the Migraine Severity Scale and the Headache Impact Test is warranted. Second, Moreover, an important inclusion criteria for our study was a formal diagnosis of migraine. However, it is possible that some of the included participants may have had concomitant medication overuse headache (MOH). However, we wanted to assess the tool in a real-world setting where patients with migraine may have medication overuse headaches or other comorbidities. Future research should assess the psychometric properties of this tool in patients with concomitant MOH. Third, our goal was to demonstrate the validity of the GAMS in real world settings that include a heterogeneous group of migraine patients. As such, we demonstrated the validity of the GAMs using our study data. However, we recognize that there might be variations in the degree of migraine severity across subgroups, which might limit the generalizability of our study findings to specific migraine populations. For example, differences between episodic and chronic migraine were noted on several questionnaires, including the GAMS questionnaire. Patients with chronic migraine were more likely to report a higher severity of headache compared to patients with episodic migraine. Future research should assess the psychometric properties of this tool in these subgroups of migraine patients. Finally, our cohort consisted of patients seen only in the Headache Clinic at one tertiary care hospital in Calgary, Alberta (catchment > 1 million), who mostly reported “moderately severe”, to “extremely severe” migraine attacks. This might have been because most patients seen in this hospital are only referred because of the severity of their illness. Future research will also need to investigate the validity of GAMS in community-dwelling persons with migraine.

## Conclusion

In summary, we demonstrate the validity of GAMS as a brief measure of patient-reported migraine severity and identified clinical and psychosocial correlates and/or mediators of patient-reported severity of migraine in a cohort of migraine patients seen in an outpatient Headache clinic. The identified determinants can help health researchers design and implement targeted interventions, appropriate support services and adequate measures to improve disability in persons with migraine. In addition, a single-item global rating scale of migraine severity may be a rapid and efficient way for clinicians to obtain information about disease severity, but requires validation in future prospective studies.

## Abbreviations

GAMS: Global Assessment of Migraine Severity
HADS: Hospital anxiety and depression scale
HADS-A: HADS-anxiety
HADS-D: HADS-depression
MIDAS: Migraine disability assessment of scale
MOH: Medication overuse headache
NEEDS: NEurological DiseasE and Depression Study
PHQ9: 9-item Patient Health Questionnaire
SD: Standard deviation
